# Exalgin

**Published:** 1890-07-05

**Authors:** 


### EXALGIN.

Though discovered by A. W. Hofmann as long ago as 1874, the physiological and therapeutic actions of exalgin were not investigated until quite recently, the credit of having introduced it into medicine being due to Drs. Dujardin-Beaumetz and Bardet. It is methyl-acetanilid. $C_6H_5.NCH_3.C_2H_3O$, derived from acetanilid $C_6H_5$ NH. $C_2H_3O$ by the substitution of one radicle methyl $CH_3$ for the H in the $NH_2$ group. Cahn and Hepp had already in 1887 tried it as an antipyretic, but abandoned it as being too dangerous. One gram killed a rabbit quickly with convulsions, and ·75 gram induced giddiness and slight convulsions in a phthisical patient; and Binet came to the same conclusion as to its unsuitability for reducing the temperature. Dujardin-Beaumetz found that doses of 0·2 to 0·4 grams to the extent of 0·8 in the 24 hours caused no nervous disturbance, though this quantity taken in one dose produced more or less vertigo. But *single* doses of 0·25 to 0·5 grams allayed pain in a remarkable manner, especially neuralgia and the severe paroxysms of "lightning pains" in tabes dorselis.

In diabetes it reduced both the urine and the sugar, but it is only as an anodyne in emergencies that it is likely to establish its position. Dr. Rabow gave it as such on 80 occasions to 30 patients in 0·25 gram doses with almost unfailing success, and Dr. Fraser makes this the maximum dose, finding even 0·03 to 0·06 sufficient in many cases. It is very valuable in toothache and earache, succeeding in the latter often when morphine has failed. In obstinate cases of sciatica 1·5 grams spread over the 24 hours may be required, but in muscular rheumatism it is by no means satisfactory.

